# A Biomimetic Multi-Component Subunit Vaccine via Ratiometric Loading of Hierarchical Hydrogels

**DOI:** 10.21203/rs.3.rs-4177821/v1

**Published:** 2024-04-25

**Authors:** Fanfan Du, Simseok A. Yuk, Yuan Qian, Michael P. Vincent, Sharan Bobbala, Tirzah M. Abbott, Hyeohn Kim, Yang Li, Haoyu Li, Sijia Yi, Baofu Qiao, Evan A. Scott

**Affiliations:** 1 Department of Biomedical Engineering, Northwestern University, Evanston, IL 60208, USA.; 2 Northwestern University Atomic and Nanoscale Characterization Experimental Center, Evanston, IL 60208, USA.; 3 Department of Chemical and Biological Engineering, Northwestern University, Evanston, Illinois 60201, USA.; 4 Department of Natural Sciences, Baruch Colleg-e, City University of New York, New York, 10010, USA.; 5 Simpson Querrey Institute, Northwestern University, Chicago, IL 60611, USA.; 6 Chemistry of Life Processes Institute, Northwestern University, Evanston, IL 60208, USA.; 7 Interdisciplinary Biological Sciences Program, Northwestern University, Evanston, IL 60208, USA.; 8 Robert H. Lurie Comprehensive Cancer Center, Northwestern University, Chicago, IL 60611, USA.

## Abstract

The development of subunit vaccines that mimic the molecular complexity of attenuated vaccines has been limited by the difficulty of intracellular co-delivery of multiple chemically diverse payloads at controllable concentrations. We report on hierarchical hydrogel depots employing simple poly(propylene sulfone) homopolymers to enable ratiometric loading of a protein antigen and four physicochemically distinct adjuvants in a hierarchical manner. The optimized vaccine consisted of immunostimulants either adsorbed to or encapsulated within nanogels, which were capable of noncovalent anchoring to subcutaneous tissues. These 5-component nanogel vaccines demonstrated enhanced humoral and cell-mediated immune responses compared to formulations with standard single adjuvant and antigen pairing. The use of a single simple homopolymer capable of rapid and stable loading and intracellular delivery of diverse molecular cargoes holds promise for facile development and optimization of scalable subunit vaccines and complex therapeutic formulations for a wide range of biomedical applications.

## Introduction

Effective vaccination relies on the ability to induce humoral and cellular immunity, with the nature of this adaptive immune response being highly dependent on the co-delivery of distinct antigen and adjuvant combinations to innate immune cells. Mimicking the diverse spectrum of immunostimulation elicited by attenuated viral and bacterial vaccines has remained a challenge for subunit vaccine formulations, which typically pair a single adjuvant and antigen for simplicity and scalability^1–3^. Furthermore, despite recent advances in creating hydrogels capable of sustained delivery of protein antigens^4–6^, approaches to combine antigens with physicochemically distinct adjuvants remain challenging. In particular for hydrophobic and charged macromolecular adjuvants in the form of lipids, small molecules, or nucleic acids, controlling the concentration and stability of each during multi-component loading within a hydrophilic network is rarely achieved^7^. To this end, nanocomposite and micelle-cross-linked hydrogels^8,9^ have been used to load hydrophobic cargoes^10^. The fabricating process for these artificial hierarchical structures is, however, tedious and the applied synthetic components are complex.

**Fig. 1.**
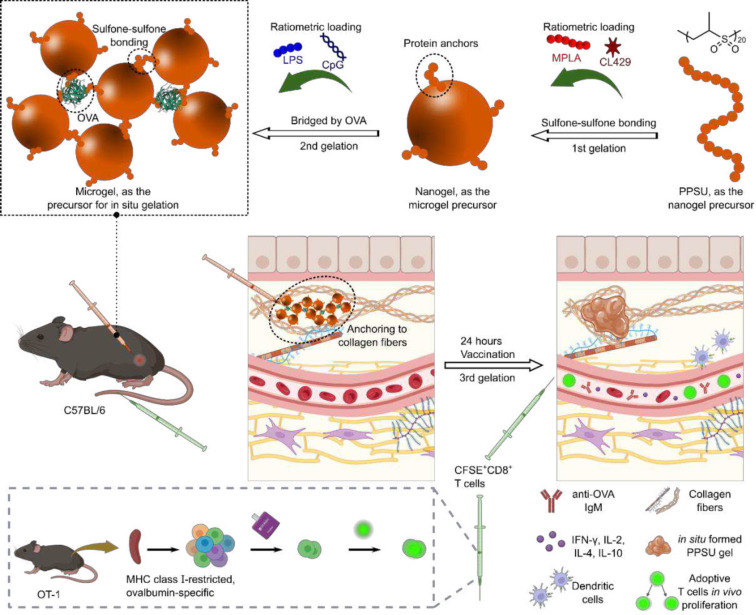
Schematic of PPSU-based hierarchical hydrogels that enable ratiometric loading of multiple adjuvants for vaccine optimization. Hydrophobic adjuvants are encapsulated through network assembly of PPSU (1^st^ gelation). Water-soluble adjuvants are adsorbed during the formation of microgels triggered by bridging nanogels with the antigen OVA (2^nd^ gelation). Microgels anchor onto collagens upon subcutaneous injection (3^rd^ gelation), allowing sustained release. After 24 h of vaccination, increases in Th1/Th2 cytokines and anti-OVA IgM antibodies result in the *in vivo* proliferation of adoptively transferred OT-1 CD8^+^ T cells.

Hierarchical materials are assembled via multi-length scale processes and frequently possess biomimetic properties that include multifunctionality, environmental responsiveness, and self-organization^11^. Here, we report on hierarchical hydrogels, assembled from a single simple homopolymer via a multi-stage process, that enable ratiometric loading and delivery of four physicochemically distinct adjuvants along with a protein-based antigen (Fig. 1). Starting with a poly(propylene sulfone) (PPSU)^12^ solution, the addition of water promotes interchain sulfone-sulfone bonding, yielding nanogels with short PPSU coronas capable of self-bonding or protein anchoring. When transferred into protein solutions in saline, the nanogels irreversibly aggregate into microgels as a secondary structure via surface fusion or protein bridging. Following subcutaneous injection, the microgels then bind to extracellular matrix (ECM) components, mainly collagen fibers, via PPSU coronas to form stable *in situ* nanocomposite hydrogels as the tertiary structure. The delivery system takes advantage of efficient loading (>90%) throughout the process, allowing us to develop an optimized vaccine formulation that consists of monophosphoryl lipid A (MPLA)^13^, CL429^14^, CpG oligodeoxynucleotides (ODN)^15^, lipopolysaccharide (LPS)^16^, and the protein antigen ovalbumin (OVA). *In vitro* and *in vivo* experiments demonstrate enhanced humoral and cell-mediated immune responses for these five-component nanogel vaccines compared to formulation with a more standard single adjuvant and antigen pair.

## Results and Discussion

### Ratiometric loading of five separate vaccine immunostimulants

The preparation of PPSU nanogels has been described in our previous publications^12,17^. As prepared, the nanogels have a zeta potential between −30 and −40 mV^12,17^ that prevents aggregation in water. Importantly, the coronas of the nanogels consist of “living” PPSU that will undergo further sulfone-sulfone bonding on demand. This PPSU corona is also capable of anchoring proteins while preserving protein bioactivities at the interfaces^17^. To form subcutaneous depots, PPSU nanogels were self-cross-linked or bridged by serum and ECM proteins following injection (**Fig. S1**). The strategy involves the addition of salts to promote corona fusion by shielding the electrostatic repulsion among nanogels, or the use of low concentration protein solutions (e.g. hemoglobin, neutral charged) in water to bridge two nanogels through surface adsorption. Although PPSU surfaces show high affinity to nonspecific proteins, the addition of salts is required to trigger the adsorption of highly negatively charged proteins^17^, such as OVA (Fig. 1a). That is, incubating PPSU nanogels in phosphate buffered saline (PBS) with OVA leads to the formation of microgels through both corona fusion and crosslinking via protein antigen bridges (Fig. 1b–c and **Fig. S2a-c**). By employing protein payloads as the crosslinking agent, the minimalist secondary microgels engineered here do not require additional cross-linking agents or stabilizers, allowing rapid and simple preparation of an injectable hydrogel depot. Furthermore, the primary nanogels support the pre-encapsulation of physicochemically distinct adjuvants given their high encapsulation efficiencies (> 95%) for a wide range of organic cargoes^12^.

**Fig. 2.**
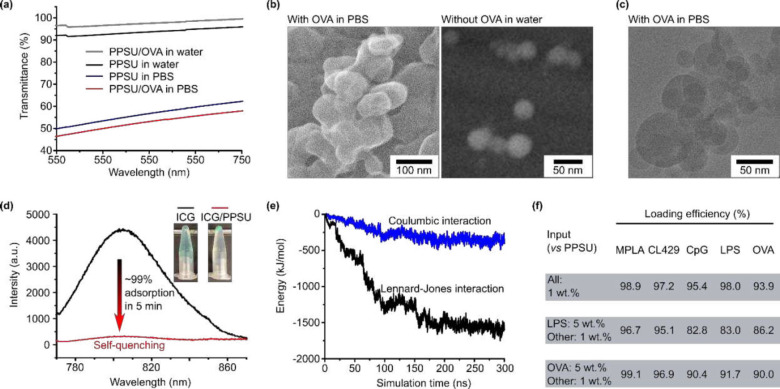
PPSU nanogels enable the ratiometric loading of multiple cargoes and subsequent assembly into microgels. **(a)** Comparison of the extinction spectra of the mixture of PPSU nanogels (1 mg/mL) with 1 wt.% of OVA in PBS or water. **(b)** CryoSEM of PPSU microgels following salt-induced gelation of nanogels via OVA bridges in PBS. **(c)** CryoTEM of PPSU microgels formed by mixing PPSU nanogels with OVA in PBS. **(d)** The complete self-quenching indicates the efficient capture of ICG during the formation of microgels in PBS. The inset shows ICG/PBS solutions before and after the addition of PPSU nanogels. The photos were taken after centrifugation. **(e)** Computational studies suggest that the PPSU surface is capable of molecular capture through hydrophobic interactions (Lennard-Jones). **(f)** Multiple adjuvants, including MPLA, CL420, CpG, and LPS can be co-loaded in a ratiometric manner while using the antigen OVA to bridge nanogels.

Amphiphiles can also to be loaded during the formation of microgels. We demonstrated, both experimentally and through simulations, the preserved affinity of PPSU surfaces to capture amphiphilic cargoes in aqueous solution during microgel formation. When we mixed PPSU nanogels with indocyanine green (ICG) in PBS, we detected by fluorescence measurements the efficient (~99%) and rapid (< 5 min) adsorption of the dye, which completely quenched the fluorescence (Fig. 2d). Near 100% adsorption of many other amphiphilic dyes at PPSU interfaces were shown in our previous report^17^. Explicit solvent all-atom molecular dynamics simulations were performed for mixtures of 10 ICG molecules and a PPSU nanogel in water, showing that all ICG molecules were adsorbed by the nanogel (Fig. 2e and **Fig. S3**). The simulation revealed that the adsorption is directed by hydrophobic interactions, consistent with our previous findings for the adsorption of proteins^17^. These results essentially suggest that efficient adsorption can be generalized to diverse therapeutics and biologics that have amphiphilic properties.

We explored the versatility and universality of PPSU by optimizing the loading of diverse adjuvants. Vesicular nanostructures (e.g. liposomes and polymersomes) typically display low encapsulation efficiency for water-soluble cargoes^18–20^. Micelles show efficient loading only for hydrophobic molecules^21^, whereas conventional hydrogels can only trap hydrophilic solutes^6^. By contrast, PPSU hydrogels showed > 90% loading efficiencies for all these mentioned types of cargoes (**Fig. S4**), including MPLA (non-ionic lipid amphiphile), CL429 (hydrophobic small molecule), CpG (hydrophilic nucleic acid), LPS (ionic lipid amphiphile), and OVA (protein). Because vaccine responses can be modulated and customized via the simultaneous delivery of multiple synergistic adjuvants^22^, we further demonstrated the ability of PPSU hydrogels to effectively co-load the antigen with all four adjuvants, showing that the resulting formulations can be easily controlled by the input ratios (Fig. 2f). Of note, these formulations mimic the full range of immunostimulation of an attenuated bacterium^23–25^.

### *In situ* formation of PPSU tertiary hydrogels upon administration

**Fig. 3.**
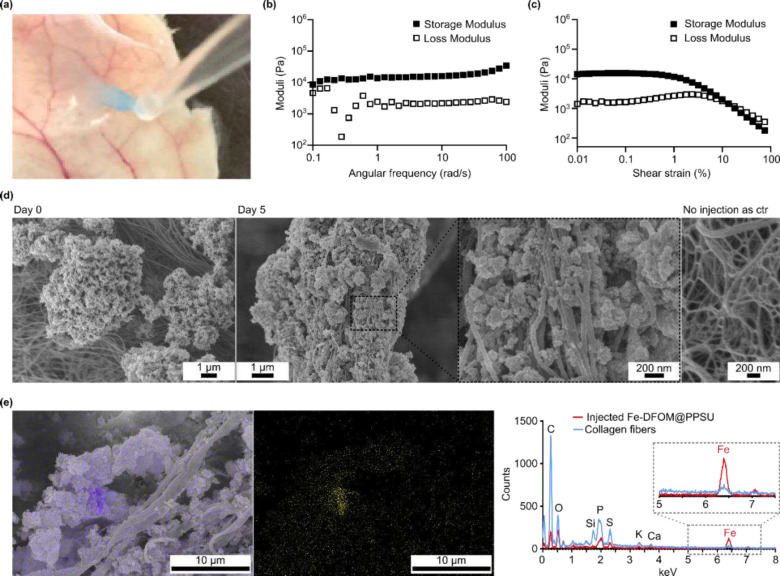
Characterization of tissue-bound hydrogels that formed following subcutaneously injection of PPSU microgels. **(a)** Dropping PPSU microgels (colored by ICG) onto an excised skin mimics the process of subcutaneous injection that anchor the microgels onto tissues. **(b)** Frequency-dependent oscillatory experiments conducted in a linear viscoelastic regime. **(c)** The storage and loss moduli across the evaluated frequency range. (b-c) Rheological measurements were performed on excised samples 30 min post injection, demonstrating the *in-situ* formation of tertiary hydrogels. **(d)** SEM images of excised gels on day 0 (30 min post injection) and 5 days post injection. The SEM image of collagen fibers is included for comparison. **(e)** EDS elemental mapping of Fe^3+^-loaded hydrogels 30 min post injection.

Because the surfaces of the microgels were not exposed to excess protein for passivation^17^, we hypothesized that surface-accessible domains would be available for subsequent tissue attachment upon subcutaneous injection. This was confirmed by a model experiment in which we dropped the suspension of PPSU microgels onto an excised skin and observed an immediate immobilization of the microgels (Fig. 3a and **Fig. S5**). We then subcutaneously injected PPSU microgels and extracted the *in situ* formed tertiary hydrogels 30 minutes after administration. Frequency-dependent oscillatory experiments conducted in a linear viscoelastic regime revealed that the gels exhibited a storage modulus comparable to the local biological tissues (Fig. 3b)^26,27^. The consistently higher maintenance of the storage modulus (G’) over the loss modulus (G”) across the evaluated frequency range indicated the solid-like properties necessary for robust hydrogel formation, thereby confirming a process of *in situ* gelation upon subcutaneous administration (Fig. 3c).

We tracked by scanning electron microscopy (SEM) imaging the *in situ* gelation of PPSU microgels within tissues after the injection (Fig. 3d and **Fig. S6**). The results showed that the microgels adhered to the surface of collagen fibers 30 minutes post administration. Adhesion of the microgels towards the collagen fibers was also verified at day 5 post injection(Fig. 3d). Fusion between PPSU and other surrounding tissues was also found (**Fig. S7**). The process of *in situ* gelation was accompanied by a decrease in the gel volume (**Fig. S8**). As a further demonstration, we loaded the microgels with Fe^3+^ for energy-dispersive X-ray spectroscopy (EDS) elemental mapping (**Fig. S9**) and confirmed SEM-EDS colocalization of Fe^3+^ that differentiated the tertiary PPSU hydrogels from collagen fibers (Fig. 3e).

**Fig. 4.**
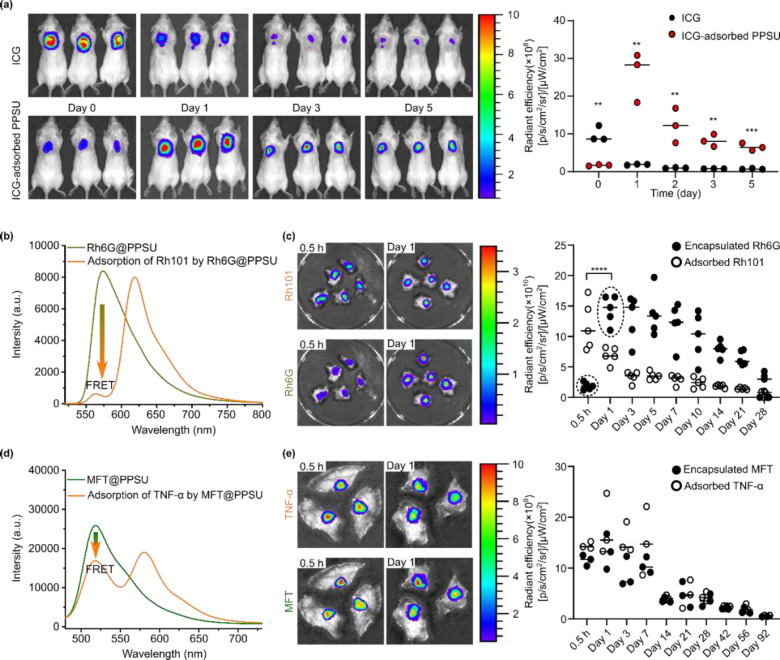
*In vivo* release of model cargoes loaded within PPSU microgels. **(a)** Real-time whole-body imaging of adsorbed ICG after SC injection showed prolonged release of hydrogel cargo. **(b)** Efficient FRET demonstrates full accessibility (within ~10 nm) of the microgel surfaces by small molecule fluorophore Rh101. **(c)** The recovery of Rh6G fluorescence at day 1 indicated quick desorption/replacement of adsorbed Rh101 upon SC injection. n = 5 mice per group. ****: p < 0.0001 by a two-sample *t*-test. **(d)** Not all of the microgel surfaces are accessible to proteins, as suggested by the inefficient FRET from encapsulated MFT in response to incubation with TNF-α. **(e)** Sustained release was achieved for the encapsulated MFT and adsorbed proteins. n = 3 mice per group.

Having obtained evidence that the tertiary hydrogels form upon subcutaneous injection, we employed the hydrogels as depots for the sustained release of diverse cargoes. Real-time whole-body imaging showed that the administration of ICG-adsorbed microgels achieved prolonged release of ICG (Fig. 4a and **Fig. S10**). Given the possibility that encapsulated cargoes could have slower release rates than their adsorbed counterparts, we proceeded to investigate the effect of our drug loading strategy on *in vivo* release kinetics. Förster resonance energy transfer (FRET)^28^ was applied as a tool in the comparison study to exclude the interference of concentration-induced fluorescence quenching. FRET imaging of the excised skins was used to monitor the *in vivo* release of a FRET pair consisting of encapsulated Rhodamine 6G (Rh6G) and adsorbed Rh101. With the fluorescence of the encapsulated Rh6G nearly completely quenched by the adsorption of Rh101 (Fig. 4b and **Fig. S11**), a burst release of Rh101 at 1 day post injection was revealed by the recovery of Rh6G fluorescence (Fig. 4c). Sustained release of the two dyes began on day 3, achieved through the liquefication of the tertiary hydrogels. These results demonstrated an erosion-controlled release process for the encapsulated dyes, whereas the release of the adsorbed dyes could be first triggered by protein replacement, a diffusion-controlled process that depends on the accessibility of nanogel surfaces. Not all the nanogel surfaces are accessible to proteins during the formation of the secondary hydrogels in PBS. This was confirmed by the inefficient FRET shown in Fig. 4d, where the adsorption of excess TNF-α only partially decreased the fluorescence intensity of the encapsulated MFT. The subsequent *in vivo* release demonstrated an erosion-controlled release process of up to 92 days for both the cargoes (Fig. 4f and **Fig. S12**), regardless of the loading strategies.

### Employing PPSU hydrogels for multi-component vaccine optimization

The sustained release system investigated here allowed us to design 5-component vaccines that typical require a tedious or impractical process for optimizing the concentration and ratio for each adjuvant. To this end, we used RAW-blue macrophages to assess retention of adjuvant bioactivity upon loading by PPSU at a low, mild, and high concentration, based on their published effective ranges (**Fig. S13**)^29–33^. The results showed that PPSU alone had no effect on RAW-blue macrophages, whereas the adjuvant-loaded hydrogels induced the activation of NF-kB. They have either equally effective or even enhanced activities compared to the free form adjuvants of LPS and CL429, respectively.

To determine the immunogenicity of the 5-component formulation (OVA-bridged PPSU microgels with four adjuvants, termed PPSU-4Ad) on antigen-presenting cells (APCs), PPSU-4Ad, CpG-loaded PPSU (PPSU-CpG), and OVA-loaded PPSU (PPSU-OVA) were incubated with primary C57BL/6 bone marrow-derived dendritic cells (BMDCs). Of note, each formulation contained OVA as the antigen. After 24 h, activation marker expression of the BMDCs, including MHC-II, CD40, CD80, and CD86, were assessed by flow cytometry. Low immunogenicity for PPSU-OVA was confirmed, as indicated by the same level of expression for all tested markers as that of the PBS group. But PPSU-4Ad strongly activated CD40, CD80 and CD86 expression on BMDCs, whereas the single adjuvant loaded group (PPSU-CpG) only marginally activated CD40 and CD86 (**Fig. S14**). Moreover, PPSU-4Ad stimulated MHC-II expression. These results verify that simultaneous delivery of antigen and adjuvants by PPSU-4Ad to APCs can induce a strong immunostimulatory response.

**Fig. 5.**
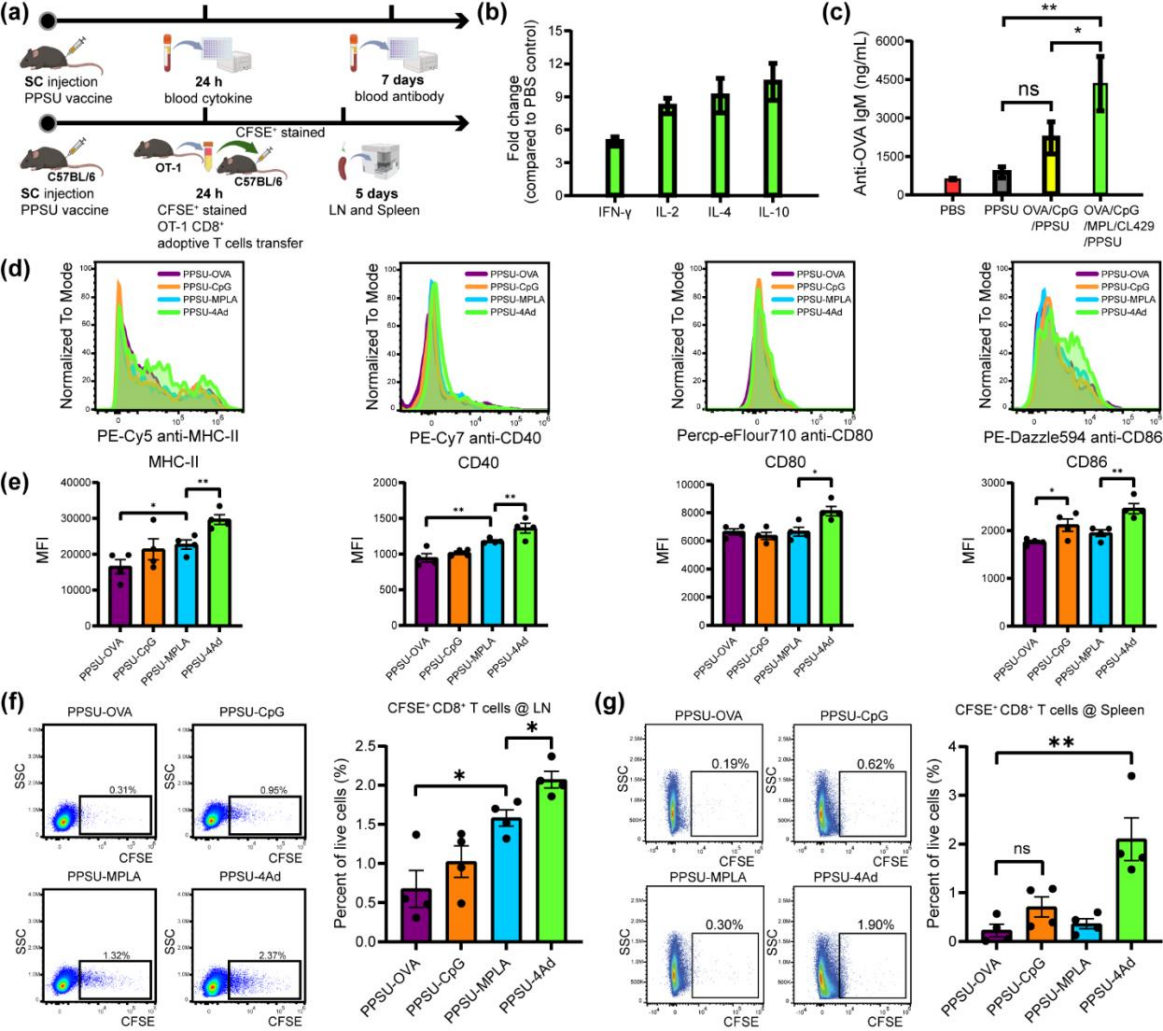
Validation of multi-adjuvant/antigen-loaded PPSU hydrogels as a subunit vaccine. **(a)** Schematic schedule of *in vivo* blood analysis and adoptive T cell transfer. (created with BioRender.com). **(b)** Cytokine fold changes 24 h after subcutaneous injection of the multi-antigen/adjuvant (LPS excluded) loaded PPSU relative to PBS control in mice. n = 3 mice per group (mean ± s.d). **(c)** Blood anti-OVA IgM antibody levels 7 days post subcutaneous administration of the multi-antigen/adjuvant (LPS excluded) loaded PPSU relative to single adjuvant, blank PPSU and PBS controls. n = 3 mice per group (mean ± s.d). **(d)** Flow cytometry histogram and **(e)** mean fluorescence intensity (MFI) of activation markers (MHC-II, CD40, CD80, and CD86) on dendritic cells in the lymph node after 4 days following the adoptive transfer of carboxyfluorescein succinimidyl ester (CFSE) stained CD8^+^ T cell from OT-1 mice to C57BL/6 mice. n = 4 mice per group (mean ± s.d). **(f)** Gating strategies and percentage of live CFSE^+^CD8^+^ T cells at lymph node and **(g)** spleen after 4 days post adoptive T cell transfer. n = 4 mice per group (mean ± s.d). *: p < 0.05, **: p < 0.01, and ns: not significant by unpaired *t*-test.

C57BL/6 mice were then immunized subcutaneously with either PPSU-CpG or PPSU-4Ad (Fig. 5a, LPS was not included as an adjuvant for cytokine and antibody experiments). Blank PPSU and PBS groups were included as controls. At 24 h post-injection, PPSU-4Ad induced both Th1 and Th2 serum cytokines (Fig. 5b). The elevated Th2 cytokine levels indicate a tendency for PPSU-4Ad to favor humoral responses^34^. Seven days post-immunization, mice immunized with PPSU-4Ad showed a significant increase in anti-OVA IgM antibodies compared to those immunized with PPSU-CpG or the blank PPSU and PBS controls (Fig. 5c).

Next, we investigated PPSU-4Ad for its ability to induce antigen-specific T cell proliferation *in vivo* using the OT-1 mouse strain (Fig. 5a). This strain has a transgenic T cell receptor designed to recognize OVA, resulting in MHC class I-restricted, OVA-specific, CD8+ T cell proliferation upon successful vaccination against OVA as an antigen^35^. C57BL/6 mice were immunized with PPSU-4Ad, single adjuvant (PPSU-CpG or PPSU-MPLA) or only antigen (PPSU-OVA) loaded hydrogels subcutaneously at the tail base. Then, CD8^+^ T cells isolated from OT-I transgenic mice were stained with Carboxyfluorescein succinimidyl ester (CFSE) dye and adoptively transferred to mice one day post immunization. 96 h later, the mice were sacrificed, inguinal lymph nodes (LNs) and spleen were harvested. First, we tested the activation marker expression on CD11C+ DCs in the LNs. Consistent with the *in vitro* results, PPSU-4Ad significantly upregulated MHC-II, CD40, CD80, and CD86 expression on CD11c^+^ DCs in the LNs (Figs. 5d–e). Moreover, significant proliferation of CFSE^+^ OT-I CD8^+^ T cells in both LNs and spleen was observed in mice immunized with PPSU-4Ad, compared to PPSU groups loaded with only a single adjuvant or antigen (Figs. 5f–g). Without additional adjuvants, the single adjuvant and PPSU-OVA groups showed a limited number of divisions *in vivo* at 4 days post-transfer, similar to the findings reported previously^36,37^. Collectively, these results verify that PPSU-based hierarchical hydrogels effectively co-deliver antigen and multiple physicochemically distinct adjuvants for synergistic immune responses following subcutaneous administration. Additionally, fine-tuning the antigen-specific immune responses is straightforward due to the capacity for ratiometric loading, achievable by varying the combinations of antigens and adjuvants.

## Conclusion

Our studies demonstrate a hierarchical hydrogel-based platform for the engineering of synthetic subunit vaccines with biomimetic, synergistic immune responses. The strategy involves ratiometric loading and tissue attachment that make use of a self-assembling homopolymer, which assembles into nanogels in water, then to microgels in saline, and ultimately to tertiary hydrogels *in vivo*. When delivering four physicochemically distinct adjuvants and a protein antigen, the hydrogels elicited vaccine-related immune responses both *in vitro* and *in vivo* that were superior to standard single adjuvant formulations. These minimalist hierarchical hydrogels, formed from a single simple homopolymer, achieves efficient ratiometric loading to simplify complex drug delivery applications where controlling multiple drug concentrations is tedious or not feasible.

## MATERIALS AND METHODS

### Chemicals and reagents.

PPSU_20_ was synthesized in our previous paper^12^. All chemicals were purchased from Sigma-Aldrich and used as received, unless otherwise stated. HBB (lyophilized powder) was obtained from Sigma (H2500; LOT# SLCB6082). Ethylenediaminetetraacetic acid disodium salt solution and albumin from chicken egg white were purchased from Sigma-Aldrich (St. Louis, MO, USA). Invitrogen^™^ eBioscience^™^ 1X RBC Lysis Buffer, Falcon^™^ Cell Strainers, Falcon^™^ Round-Bottom Polystyrene Test Tubes with Cell Strainer Snap Cap 5mL, Propylene Sulfide (stabilized with Butyl Mercaptan) 98.0+%, Heat-Inactivated Fetal Bovine Serum (FBS), Gibco Dulbecco’s Modified Eagle’s medium (DMEM), Gibco Roswell Park Memorial Institute 1640 medium (RPMI), Gibco Dulbecco’s Phosphate Buffered Saline (without calcium chloride and magnesium chloride), hexamethyldisilazane (HMDS), Th1/Th2 cytokine ELISA kit (Invitrogen), Hyaluronidase Type I-S from Bovine, Ethanol (200 Proof), Indocyanine Green, carboxyfluorescein succinimidyl ester (CFSE), and Methotrexate Fluorescein Triammonium Salt were purchased from Thermo Fisher Scientific (Waltham, MA, USA). Lipopolysaccharide (LPS), CL429, CpG oligodeoxynucleotide, Class C (CpG-ODN), and monophosphoryl lipid A (MPLA) were purchased from InvivoGen (San Diego, CA, USA). Collagenase, Type 4 and Deoxyribonuclease I were purchased from Worthington Biochemical (Lakewood, New Jersey, USA). Mouse Anti-OVA IgM Antibody Assay Kit was purchased from Chondrex, Inc (Woodinville, WA, USA). Flow cytometry cell staining buffer (1x), anti-mouse CD16/CD32, fixable zombie NIR viability dye and all antibody cocktail (PE-Cy5 anti-mouse MHC-II, PE-Cy7 anti-mouse CD40, APC anti-mouse CD80, Percp-eFlour710 anti-mouse CD80, APC-Cy7 anti-mouse CD11c, PerCP-Cy5.5 anti-mouse CD45, BV510 anti-mouse CD3 and PE-Dazzle594 anti-mouse CD86) were purchased from BioLegend (San Diego, CA, USA). BD Veo U-100 Syringes 31g 3/10cc 6mm 90 Count was purchased from ADW Diabetes (Pompano Beach, FL, USA). STEMCELL CD8a+ T Cell Isolation Kit was purchased from STEMCELL Technologies (Vancouver, BC, Canada). InVivoMAb anti-mouse TNF-α and InVivoPure pH 7.0 Dilution Buffer were purchased from Bio X Cell (Lebanon, NH, USA).

### Preparation of PPSU hydrogels.

200 μL of PPSU in DMSO (25 mg/mL; with or without co-dissolved cargoes) were thoroughly mixed with 200 μL of water. The obtained 400 μL of mixtures were further thoroughly mixed with 200 μL of water. Dialysis in water was then performed to completely remove DMSO. The obtained PPSU nanogels were stocked at 4 °C for further use. PPSU microgels were prepared by mixing 900 μL of PPSU nanogels (5 mg/mL in water; with or without co-dissolved antigen or adjuvants) with 100 μL of PBS (10 ×). After 5-min incubation, the mixture was centrifuged, and the microgel pellet was collected by discarding the supernatant. The pellet was resuspended in fresh PBS, followed by repeating the centrifugation-resuspension cycle for a total of three times to remove unbound antigen or adjuvants. The microgels were resuspended in PBS (10 mg/mL) for injection.

### Animals.

All procedures involving animals received approval from Northwestern University’s Institutional Animal Care and Use Committee, adhering to the NIH guidelines for the ethical treatment and utilization of laboratory animals. Female BALB/c mice, C57BL/6 mice, and C57BL/6-Tg(TcraTcrb)1100Mjb/J (OT-1, JAX stock #003831) mice, all aged 6–8 weeks, were procured from the Jackson Laboratory in Bar Harbor, Maine, USA.

### Fluorescence measurement.

Fluorescence spectra were recorded by an RF-6000 spectrofluorometer using LabSolutions RF software.

### TEM imaging.

Negative staining was used for imaging the samples. To prepare the staining reagent, 1.5% uranyl formate (UF) was dissolved in water. The pH was then adjusted to 4.5 by the addition of 10 M KOH. Forrmvar carbon film grids (FCF400-CU; Electron Microscopy Sciences) were treated for 20 s in a Pelco easiGlow glow discharger (0.24 mbar; 15 mA). A pretreated grid was suspended upside down in 20 μL of hydrogel suspension (0.7 mg/mL) for 30 s. The sample-loaded grid was further suspended upside down in 30 μL of 1.5% UF twice for 15 s, followed by the removal of excess UF solution by blotting with Whatman filter paper. TEM Images were acquired at 30 k on a JOEL 1400 TEM operating at 120 kV.

### Cryo-TEM imaging.

Fresh samples were prepared by applying 3 μL of suspension on pretreated (same as TEM imaging) holey lacey carbon 400 mesh TEM copper grids (Electron Microscopy Sciences). Following a blot of 3 s, the grids were plunge-frozen (Gatan Cryoplunge 3 freezer). Images were acquired using a field emission transmission electron microscope (JEOL 3200FS) operating at 300 keV. Digital Micrograph software (Gatan) was used to align the individual frames of each micrograph to compensate for stage and beaminduced drift.

### Cryo-SEM imaging.

300-mesh Cu grids with a carbon membrane were glow-discharged for 30 s in a Pelco easiGlow glow-discharger at 15 mA with a chamber pressure of 0.24 mBar. 5 uL of samples were pipetted onto a grid, and briefly blotted by hand while paying careful attention to make sure the liquid layer did not dry before plunging the grid into liquid ethane. Grids were then loaded into a Gatan 626.5 cryo transfer holder and viewed in a Hitachi HD2300 STEM with a field emission source at 200 kV utilizing an SE detector. Prior to gathering image data, the cryo transfer holder was warmed from −180 °C to −145 °C at a rate of 5 °C/minute. This warming process slowly sublimed away background ice under vacuum to reveal particles.

### All atom explicit solvent molecular dynamics simulations.

The 125-chain PPSU nanoparticle was obtained by the previous simulations^17^. Indocyanines (ICG) molecule was built with the package Avogadro^38^. The CHARMM force field parameter for ICG was generated with CGenFF^39^. After the simulation of 125-chain nanoparticle approached equilibrium, 10 molecules of ICG were solvated into the system along with the counter-ions (10 Na^+^). The ICG molecules were distributed randomly in the simulation box. Energy minimization, NVT equilibration, NPT equilibration, and 200 ns molecular dynamics production run were performed with the same simulation parameters aforementioned. The binding energy between ICGs and the nanoparticle was calculated with the *gmx energy* tool.

### Loading efficiency.

2,3-cGAMP (Invivogen) was quantified by liquid chromatography-mass spectrometry. CL429 (Invivogen) was quantified by a colorimetric assay that forms metal-complex with the absorbance at 660 nm. The loading efficiencies of Alexa-647-labelled OVA (Invitrogen; *λ*_em_/*λ*_ex_ = 668/650 nm), FITC-labelled CpG-ODN (Invivogen; *λ*_em_/*λ*_ex_ = 525/495 nm), and cy3-labelled LPS (Nanocs; *λ*_em_/*λ*_ex_ = 570/550 nm) were determined by fluorescence measurements.

### Rheological characterization.

200 μL of hydrogels (10 mg/mL in PBS) was subcutaneously administered to the neck. After 30 minutes, the injection site was excised, and the hydrogel was retrieved from the skin. Rheological analysis was conducted at 37 °C in a humidified atmosphere using modular compact rheometer (Anton Paar MCR302). All measurements were performed using an 8 mm parallel plate geometry with a 0.3 mm gap height. An amplitude sweep was performed at a constant angular frequency of 6.28 rad s^−1^ to verify the linear viscoelastic regime. Frequency dependence of the storage and loss moduli was analyzed in oscillatory mode in the linear viscoelastic regime (0.1%).

### SEM-EDS analysis.

The nanogels were loaded with 5 wt.% of deferoxamine mesylate (DFOM). Excess FeCl3 was added to induce microgel formation and coordination. The hydrogels were centrifuged, and the pellet was collected. The pellet was resuspended in PBS, followed by centrifugation. This step was repeated a total of three times to prepare FeCl_3_-loaded hydrogels. Before injection, the C57BL/6 female mouse neck area was shaved, and remaining hair was removed using Nair. A subcutaneous injection of 200 μL of the FeCl_3_-loaded hydrogels (10 mg/mL in PBS) was administered to the neck. After 30 minutes, the skin along with hydrogels was collected for SEM analysis. A 2.5% glutaraldehyde solution was applied for fixation for two hours at room temperature (glutaraldehyde was diluted from 25% with 0.1 M sodium cacodylate buffer, which was further diluted from 0.4 M sodium cacodylate buffer with water). The skins with hydrogels were then rinsed three times with 0.1 M sodium cacodylate buffer and once with water (each rinse lasting 10 minutes) and incubated in 2% osmium tetroxide for 30 minutes. After another three rinses with water (each lasting 10 minutes), the sample underwent dehydration with a series of ethanol gradients (30%, 50%, 70%, 85%, and 95%) for 10 minutes at each step. The dehydrated cells were rinsed with 100% ethanol two times for 10 minutes each and were immersed in 1:1 mixture of HMDS and ethanol for 30 min and were subsequently rinsed twice with 100% HMDS for 30 min each. After overnight drying, the cells were placed on a carbon tape attached to an aluminum stud and coated with SPF Osmium Coater (21 nm thickness) for SEM-EDS analysis. The morphology and Fe content were then observed using a Hitachi SU-8030 C-FEG SEM and Oxford Instrument X-Max 80 energy dispersive x-ray spectroscopy (EDS) detector. Due to the electron beam sensitive of the sample, a beam energy of 15 kV was used for x-ray microanalysis to see the Fe Kα peak at 6.4 eV. EDS spectral maps were collected for several areas of interest to determine the spatial distribution of elements. Areas concentrated in Fe were then probed individually to determine the presence and relative wt.% Fe in those areas.

### *In vivo* release.

Either 20 μg of ICG (*λ*_em_/*λ*_ex_ = 800/745 nm) or 20 μg of ICG adsorbed by 2 mg of hydrogels in 200 μL was subcutaneously administered to the neck of Female BALB/c mice. Live monitoring of ICG was conducted at different time points: 0, 1, 2, 3, and 5 days. For FRET analysis, 0.5 wt.% of Rh6G were encapsulated in PPSU nanogels, followed by the adsorption of 0.5 wt.% of Rh101. 200 μL of the resulting hydrogel suspension in PBS (10 mg/mL of PPSU) was subcutaneously administered to the neck of Female BALB/c mice. The mouse was euthanized at various time points, including 0.5 hours, 1, 3, 5, 7, 10, 14, and 21 days. The skins containing hydrogels were excised and imaged using IVIS Lumina (*λ*_em_/*λ*_ex_ = 465/640 nm for Rh101, *λ*_em_/*λ*_ex_ = 465/560 nm for Rh6G). Similarly, 2 wt.% of fluorescein-labeled methotrexate (MFT) were encapsulated in PPSU nanogels, followed by the adsorption of excess TNF-α (labeled with tetramethylrhodamine isothiocyanate) in PBS. The unbound TNF-α was removed. 200 μL of the resulting hydrogel suspension in PBS (10 mg/mL of PPSU) was subcutaneously administered to the neck of Female BALB/c mice. The mouse was euthanized, then the skin was excised at various time points, including 0.5 hours, 1, 3, 7, 14, 21, 28, 42, 56, and 92 days for IVIS analysis. Images were then analyzed using IVIS Lumina (*λ*_em_/*λ*_ex_ = 465/600 nm for TNF-α, *λ*_em_/*λ*_ex_ = 465/520 nm for MFT). The v.4.5.5, PerkinElmer Image Software was used for IVIS.

### *In vitro* adjuvant activity assay.

NF-kB/AP-1 activation was assessed in RAW-blue macrophages (Invivogen) following the manufacturer’s protocols. Cells were washed twice with PBS before use. RAW-blue cells (180 μL) in media (DMEM supplemented with 10% heat-inactivated FBS and 1% Pen/strep) were seeded at a density of 10^5^ cells/well in 96-well plates. Samples with varying concentrations in a PBS solution (20 μL) were added to each well. After 24 hours, 50 μL of culture supernatants were collected and incubated with 150 μL of QUANTI-Blue substrate (Invivogen) for 2 hours. The level of secreted embryonic alkaline phosphatase (SEAP) was determined using an M3 plate reader (SpectraMax) at an absorbance of 655 nm.

### *In vivo* blood analysis.

C57BL/6 mice were subcutaneously immunized with either 100 μL of OVA-CpG PPSU or OVA-CpG-MPL-CL429 mult-adjuvant PPSU. Controls included mice injected with blank PPSU and PBS only. Each mouse received a dosage of 30 μg of OVA, 20 μg of CpG, 25 μg of MPL, and 25 μg of CL429. At 24 hours post-injection, blood samples were collected, and serum cytokines were quantified using a Th1/Th2 cytokine ELISA kit. Seven days post-immunization, all mice were euthanized, and whole blood was obtained for the analysis of serum anti-OVA IgM antibodies using a Mouse Anti-OVA IgM Antibody Assay Kit.

### Mouse primary bone-marrow-derived dendritic cells (BMDCs).

Bone marrow cells were harvested from the isolated tibias and femurs of C57BL/6 mice. The procedure involved removing muscles and connective tissues from the bone, followed by flushing the cut ends of the bones using a syringe filled with complete RPMI-1640 medium (supplemented with 10% heat-inactivated FBS and 1% Pen/strep). The bone marrow suspensions were transferred into a sterile 15 mL centrifuge tube and centrifuged at 400 × *g* for 5 minutes. Subsequently, the suspended cells were resuspended in complete RPMI-1640 medium supplemented with GM-CSF (20 ng/mL). The cells were plated at a density of 2 × 10^6^ cells per well in 6-well plates and incubated at 37 °C in a 5% CO2 humidified incubator for 6 days. Half of the medium was replaced with fresh GM-CSF-containing medium on day 3. After 6 days, non-adherent and loosely adherent cells were treated with 100 μg of PPSU that contained 100 ng of MPLA, 100 ng of CL429, 100 ng of LPS, 100 ng of CPG-ODN, 10 μg of OVA and incubated for an additional 24 hours for flow cytometry analysis. All PPSU Groups contained OVA protein (1 wt.% of PPSU). The mean fluorescence intensity (MFI) was quantified by flow cytometry using a 3L 16V-14B-8R Aurora flow cytometer (CyTek). Spectral unmixing was completed using SpectroFlo (CyTek), followed by the analysis using FlowJo software.

### OT-1 CD8^+^ adoptive T cell *in vivo* proliferation.

C57BL/6 mice were immunized with the same dose as the one administered to BMDCs. CD8+ OT-1 T cells were isolated from the spleens of C57BL/6-Tg(TcraTcrb)1100Mjb/J mice using the STEMCELL CD8a+ T Cell Isolation Kit (STEMCELL Technologies), following the manufacturer’s instructions. Purified OT-1 T cells were labeled with a cell proliferation dye, carboxyfluorescein succinimidyl ester (CFSE, Thermo Fisher Scientific), according to the manufacturer’s recommended protocol. After labeling, the cells were washed and resuspended in sterile PBS. Labeled OT-1 T cells (1 × 10^6^ cells in 100 μL of PBS) were intravenously injected into recipient OT-1 mice via the tail vein. After 96 hours, mice were euthanized, and spleens and lymph nodes were harvested for further flow cytometry analysis (same procedure as prior BMDCs experiments).

### Statistical analysis.

Statistical analysis of all data was carried out using GraphPad Prism 10.1.2 (La Jolla, CA). Statistical significance was performed by unpaired *t*-test or one/two-way ANOVA followed by the recommended multiple comparisons test. A *p-*value of < 0.05 was considered statistically significant.

## Data Availability

The main text and supplementary materials contain all the available data.
